# Supplementary material for: Application of 5-Methylcytosine DNA Glycosylase to the Quantitative Analysis of DNA Methylation
Source: Int J Mol Sci. 2021 Jan 22;22(3):1072. doi: 10.3390/ijms22031072 (PMC7865733; doi:10.3390/ijms22031072)
Supplement: Supplementary file 1 [file ijms-22-01072-s001.pdf]

Supplementary Information for

Application of 5-methylcytosine DNA glycosylase to the quantitative analysis of DNA methylation

Woo Lee Choi, Young Geun Mok, and Jin Hoe Huh

Correspondence: Jin Hoe Huh
E-mail: huhjh@snu.ac.kr

This PDF file includes:

Supplementary Table S1

Table S1. List of primers for PCR amplification

Primer	Sequence (5’→3’)	Region of interest
**DME-PCR of *Hpa* II-methylated yeast genomic DNA**		
**F1a**	TACTCATCTATGGCAATTCTGAGG	
**F1b**	GGACTACAGATTACAGGTCCTTC	*Hpa* II site-methylated region
**R1**	CAGAAATATCATTTTCCAGTTTGACC	
**F2**	CAGCCAAAAAAAGTCCTTATCACC	*Hpa* II site-deficient region
**R2**	TCAGATGATCTATTTGAATCCTCTG	
**DME-qPCR of methylated pUC19 plasmid**		
**DG1764**	TAAGGGATTTTGGTCATGAGATTATC	CG site-deficient UMR
**DG1882**	GGTAACTGTCAGACCAAGTTTACTC	
**DG1883**	CACATTAATTGCGTTGCGCTCAC	M.*Sss* I-methylated HMR
**DG1884**	GGATAACCGTATTACCGCCTTTGAG	
**DG1915**	GATACGGGAGGGCTTACCATC	*Hpa* II-methylated LMR
**DG1916**	ACTGGCGAACTACTTACTCTAGCTTC	
**Bisulfite sequencing of *FWA* allele in *Arabidopsis***		
**DG662**	TAAGAGAATTAAAATGYAAAYTTTGYAAATA	Unmethylated gene *ASA1*
**DG663**	CCTAACAATRCAATCTRTTTCRAAAATRAC	
**5’-FWA F**	GGTTYTATAYTAATATYAAAGAGTTATGGGYYGAAG	5’-regulatory region upstream from start codon of *FWA* gene [1]
**5’-FWA R**	CAAARTACTTTACACATAARCRAAAAACARACAAATC	
**DME-qPCR of *FWA* allele in *Arabidopsis***		
**DG1287**	GAACCCTTTTAAAGGACTAGCCCA	Unmethylated gene *ASA1*
**DG1288**	CGAAAATGACTGTTAAGAGCTCGTTG	
**DG1289**	CATACGAGCACCGCTTTACGG	5’-regulatory region 0.7 kb upstream from the start codon of the *FWA* gene
**DG1290**	GAACCAAAATCATTCTCTAAACAAAGTGT	
**CAPS marker**		
**DG660**	CCAGCCAAATATCAGATCTTGCGCC	5’-regulatory region from the start codon of the *FWA* gene
**DG661**	CCTTAAACAACCAAATAGCASTCCGACCAATG	

(Continued)

**Primer**	**Sequence (5'→3')**	**Region of interest**
**Bisulfite sequencing of *Cnr* epiallele**		
**ATP1-1 F**	TGAAYGAGATTYAAGYTGGGGAAATGGT	Unmethylated gene *ATP 1* [2]
**ATP1-1 R**	CCCCCTTCCATCAATARRTACTCCCA	
**DG1680**	TTYTAAGYTGYTAYAGAGATATTGGAAGAG	*Cnr* epiallele 2.5 kb upstream from the start codon of the *CNR* gene [3]
**DG1682**	CARATTAAAACACTTCATCAATTRAAACAA	
**DME-qPCR of *Cnr* epiallele**		
**DG1801**	GAATGAGAATGTAGGGATTGTTGTC	Unmethylated gene *ATP* 1
**DG1802**	CCCTTCCATCAATAGGTACTCCC	
**DG1803**	CAACAGCTATCATTTATTATGTTACCTC	*Cnr* epiallele 2.5 kb upstream from the start codon of the *CNR* gene
**DG1804**	AAATCATGTAATATAGCTAGGCAGAGG	
**RT-qPCR of *CNR***		
**CAC F**	CCTCCGTTGTGATGTAACTGG	SGN-U314153 (*CAC*) gene [4]
**CAC R**	ATTGGTGGAAAGTAACATCATCG	
**TIP41 F**	ATGGAGTTTTTGAGTCTTCTGC	SGN-U321250 (*TIP41*) gene [4]
**TIP41 R**	GCTGCGTTTCTGGCTTAGG	
**QRT-PCR forward primer**	TCCGGGAATTGACAGAAGATAGAGAG	*CNR* gene [3]
**QRT-PCR reverse primer**	TTCTGTCAGCAATGTAGCAGATTTCA	
**Bisulfite sequencing of the DMR in the *CNR* promoter**		
**DG2395**	AGTGGAAGGAYAATTYTAAATTATTTYTGA	Unmethylated gene *Actin* (Solyc01g066800)
**DG2396**	CTAAATCCAACRAAATRCCTAARCCCTATAC	
**DG2364**	GAGGAGTTGTGTYAAYYATAGAYYAAAGYYAGTGG	DMR 0.7 kb upstream from the start codon of the *CNR* gene [5]
**DG2365**	CACTCTACTRRACCRACATRRACAACTRARARACCAAC	
**DME-qPCR of the DMR in the *CNR* promoter**		
**DG2397**	CATTATAACTTGTTCATTCTTGAATCAAG	Unmethylated gene *Actin* (Solyc01g066800)
**DG2398**	TATACACGACCCCCTCCGATGTAC	
**DG2399**	GTTTAGTCGACTCCCTTCGGAATC	DMR 0.7 kb upstream from the start codon of the *CNR* gene
**DG2401**	CTCTTTTCCTCTAGCTTTGGAGGGATC	

References

1. Schoft, V.K.; Chumak, N.; Choi, Y.; Hannon, M.; Garcia-Aguilar, M.; Machlicova, A.; Slusarz, L.; Mosiolek, M.; Park, J.S.; Park, G.T.; et al. Function of the DEMETER DNA glycosylase in the *Arabidopsis thaliana* male gametophyte. *Proc. Natl. Acad. Sci. USA* **2011**, *108*, 8042–8047, doi:10.1073/pnas.1105117108.
2. Wang, J.; Wang, C.; Long, Y.; Hopkins, C.; Kurup, S.; Liu, K.; King, G.J.; Meng, J. Universal endogenous gene controls for bisulphite conversion in analysis of plant DNA methylation. *Plant Methods* **2011**, *7*, 39, doi:10.1186/1746-4811-7-39.
3. Manning, K.; Tor, M.; Poole, M.; Hong, Y.; Thompson, A.J.; King, G.J.; Giovannoni, J.J.; Seymour, G.B. A naturally occurring epigenetic mutation in a gene encoding an SBP-box transcription factor inhibits tomato fruit ripening. *Nat. Genet.* **2006**, *38*, 948–952, doi:10.1038/ng1841.
4. Exposito-Rodriguez, M.; Borges, A.A.; Borges-Perez, A.; Perez, J.A. Selection of internal control genes for quantitative real-time RT-PCR studies during tomato development process. *BMC Plant Biol.* **2008**, *8*, 131, doi:10.1186/1471-2229-8-131.
5. Zhong, S.; Fei, Z.; Chen, Y.R.; Zheng, Y.; Huang, M.; Vrebalov, J.; McQuinn, R.; Gapper, N.; Liu, B.; Xiang, J.; et al. Single-base resolution methylomes of tomato fruit development reveal epigenome modifications associated with ripening. *Nat. Biotechnol.* **2013**, *31*, 154–159, doi:10.1038/nbt.2462.
